# High prevalence of adrenal imaging abnormalities in Cushing’s disease

**DOI:** 10.1007/s12020-025-04403-8

**Published:** 2025-09-04

**Authors:** Efrat Markus, Yaron Rudman, Shlomit Koren, Ilan Shimon

**Affiliations:** 1 Endocrine and Diabetes Institute, Shamir Medical Center, Zerifin, Be’er Ya’akov, Israel; 2 Gray Faculty of Medical & Health Sciences, Tel Aviv University, Tel Aviv, Israel; 3 Institute of Endocrinology, Beilinson Hospital, Rabin Medical Center, Petah Tikva, Israel

**Keywords:** Adrenal, Cushing, Incidentaloma, Nodular lesion, Hyperplasia

## Abstract

**Introduction:**

Cushing’s disease (CD) is the most common cause of endogenous Cushing’s syndrome. Adrenocorticotropic hormone (ACTH) has trophic and mitogenic effects on the adrenal cortex that may cause diffuse adrenal enlargement and nodular lesions.

**Aim:**

To evaluate the prevalence of adrenal structural abnormalities in patients with CD.

**Methods:**

Retrospective cohort study. We conducted a computerized search in our medical centers databases for the diagnosis of CD recorded between the years 1995–2024. Out of 124 patients with ACTH dependent Cushing’s syndrome, we identified 68 patients with CD who underwent adrenal imaging. We analyzed the clinical, biochemical, and imaging data.

**Results:**

Our cohort included 68 patients (51 females, 75.0%; mean age at the time of adrenal imaging, 44.6 ± 14.9 years). Sixteen (23.5%) patients had an adrenal nodule ≥10 mm (median size, 27.5 mm, IQR 14.3–38.3), and 19 others (27.9%) had adrenal hyperplasia or nodules <10 mm. The prevalence of adrenal nodules increased with age from 16.7% in patients aged 26–35 years to 26.3% in those aged above 55. Patients with adrenal nodules were older compared to those with normal adrenal glands (mean age, 49.0 ± 12.4 vs 39.1 ± 14.9 years; *p* = 0.03), and had lower ACTH level (0.7 x ULN, IQR 0.5–1.2, vs 1.2 x ULN, IQR 0.9–1.8, *p* = 0.02).

**Conclusions:**

We identified abnormal adrenal imaging in 51.5% of patients with CD. The prevalence of adrenal nodules in our study was 10-fold higher than in the normal population, for all age groups. This suggests that chronic ACTH secretion in CD is associated with adrenal nodules appearance.

## Introduction

Cushing’s disease (CD) is the most common etiology (70%) of endogenous Cushing’s syndrome [1]. CD is caused by a pituitary adenoma that autonomously secretes adrenocorticotropic hormone (ACTH), leading to cortisol overproduction and secretion from the adrenal cortex [2].

ACTH is a predominant trophic factor of the adrenal cortex. Several animal models, in which ACTH or its receptor (melanocortin 2 receptor, MC2R) were eliminated, have confirmed the central role of ACTH in maintaining normal growth of the adrenal cortex [3, 4]. While ACTH also exerts a mitogenic effect, the precise mechanism by which it promotes adrenocortical growth and proliferation is complex and only partially understood [5]. Due to the effects of ACTH on the adrenal cortex, patients with ACTH-dependent Cushing’s syndrome have high prevalence of adrenal hyperplasia, reaching up to 60% [6, 7].

Adrenal incidentaloma (AI) is an adrenal mass (≥1 cm) detected on imaging not performed for a suspected adrenal disease [8]. Autopsy studies suggest that the overall prevalence of adrenal masses ranges from 1.1–8.7%, which increases with age [9]. In recent decades, with advancement in imaging technologies, radiological studies have become more common and accurate, with the prevalence of AI’s in imaging studies approaching values similar to those found in autopsy studies [8, 10–14].

In contrast to adrenal hyperplasia, there are limited studies examining the prevalence of adrenal nodules in CD. These studies, which included small cohorts of up to 40 patients with CD, found a significantly higher prevalence of adrenal nodules, with rates ranging from 4% to nearly 40% among studied individuals [6, 7, 15].

Our aim was to study the morphology of the adrenal glands and assess the prevalence of abnormal adrenal findings, including hyperplasia and adrenal nodules, in a large cohort of patients with CD.

## Materials and methods

We conducted a computerized search in Rabin and Shamir Medical Centers databases for the diagnosis of Cushing’s syndrome and screened for patients with ACTH-dependent Cushing’s syndrome. Cushing’s syndrome was diagnosed in patients with characteristic symptoms and signs and hypercortisolemia. Hypercortisolemia was confirmed according to laboratory findings including high 24 h urinary free cortisol (UFC), elevated midnight salivary cortisol, and abnormal 1 mg dexamethasone suppression test. We further classified these patients to ACTH-dependent Cushing’s syndrome, based on ACTH level in the normal or above the normal range.

A diagnosis of CD was confirmed by a pituitary adenoma of ≥6 mm depicted by seller MRI, an inferior petrosal sinus sampling (IPSS) supporting a pituitary source of ACTH secretion, immunostaining for ACTH and/or T-PIT in resected tumor specimens, and/or hormonal remission following successful trans-sphenoidal adenoma resection.

After identifying all patients with CD, our cohort included only patients with CD who have undergone abdominal computed tomography (CT) or abdominal magnetic resonance imaging (MRI), during the active phase of their disease.

In addition, we assembled a cohort of patients with ACTH-dependent Cushing’s syndrome in whom the source of ACTH secretion was not identified, that is, patients with ACTH-dependent Cushing’s syndrome without tumor localization.

Patients with malignant pituitary tumor and those with confirmed ectopic ACTH secretion were excluded. Patients that were treated with glucocorticoids were also excluded.

Based on an imaging report from an expert radiologist, all adrenal images were classified into three categories: normal, hyperplastic, or nodular adrenal glands. All adrenal nodules were classified according to nodule size: maximal nodule diameter below 10 mm, or ≥10 mm.

The study was approved by the Rabin Medical Center and Shamir Medical Center institutional review boards with waiver of patient consent, as complied with the Helsinki Declaration.

The authors received no funding for performing this study.

### Statistical analysis and plan

Statistical analysis was performed using IBM SPSS version 29.0 (IBM Corp., Armonk, NY).

Continuous variables were presented by Mean (SD) or Median (IQR) as appropriate. Dichotomous variables were presented by N (%).

T-test and Mann–Whitney tests were used to compare values of normally and non-normally distributed continuous variables, with Chi-Square test used for comparison of categorical variables.

Two-sided P-values less than 0.05 were considered statistically significant.

## Results

**Fig. 1:**
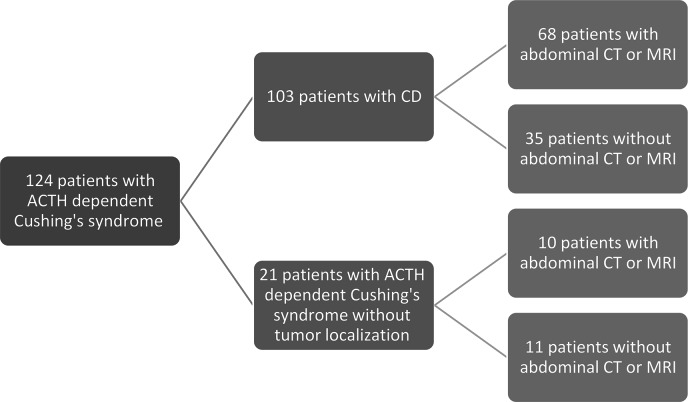
Patient selection flowchart. ACTH adrenocorticotropic hormone, CD Cushing’s disease, CT computed tomography, MRI magnetic resonance imaging

From March 1995 to December 2024, a total of 124 patients with ACTH-dependent Cushing’s syndrome were identified. There were 103 patients with the diagnosis of CD, and 21 patients with ACTH-dependent Cushing’s syndrome without tumor localization. After carefully reviewing each case, we excluded 35 patients without reported adrenal imaging (Fig. 1).

### Baseline characteristics of patients

The main cohort included 68 patients with CD and available adrenal imaging (51 females, 75.0%; mean age at the time of adrenal imaging, 44.6 ± 14.9 years). The median pituitary adenoma size (i.e. largest adenoma diameter) was 6 mm (IQR 4.75–10.25). The median ACTH level was 1.2 x ULN (IQR 0.8–1.9), and the median UFC level was 3.5 x ULN (IQR 2.0–6.0) (Table 1).

**Table 1:** Baseline characteristics of 68 patients with Cushing’s disease and 10 subjects with ACTH dependent Cushing’s syndrome without tumor localization

Variable	CD (*N* = 68)	ACTH dependent Cushing’s syndrome without tumor localization (*N* = 10)
Female – n (%)	51 (75%)	7 (70%)
Age at CT/MRI scan, years - mean (SD)	44.6 (14.9)	58.4 (15.1)
Pituitary adenoma diameter in mm, median (IQR)	6.0 (4.7–10.3)	4 (3–4)
TSS – n (%)	65 (95.6%)	0 (0%)
Post operative remission – n (%)	46 (70.8%)	
Repeat TSS – n (%)	17 (26.2%)	
Medical treatment – n (%)	39 (57.4%)	8 (80%)
Adrenal steroidogenesis inhibitors – n (%)	36 (52.9%)	7 (70%)
Somatostatin or dopamine receptor agonists– n (%)	14 (20.6%)	4 (40%)
Timing of medical treatment –		
Before TSS – n (%)	7 (17.9%)	8 (100%)
After TSS – n (%)	31 (79.5%)	0 (0%)
Before and after TSS – n (%)	1 (2.6%)	0 (0%)
Radiation therapy – n (%)	15 (22.1%)	0 (0%)
ACTH level x ULN, median (IQR)	1.2 (0.8–1.9)	1.3 (0.8–1.7)
UFC level x ULN, median (IQR)	3.5 (2.0–6.0)	3.0 (2.0–3.5)
Normal adrenal glands – n (%)	33 (48.5%)	1 (10%)
Adrenal nodule ≥10 mm – n (%)	16 (23.5%)	3 (30%)
Adrenal hyperplasia and/or nodule <10 mm – n (%)	19 (27.9%)	6 (60%)

*ACTH* adrenocorticotropic hormone, *CD* Cushing’s disease, *CT* computed tomography, *MRI* magnetic resonance imaging, *TSS* transsphenoidal surgery, *UFC* urinary free cortisol, *ULN* upper limit of normal

Sixty-five (95.6%) patients underwent trans-sphenoidal surgery (TSS), 44 of them (67.7%) had a resected adenoma expressing ACTH and/or T-PIT, 7 (10.8%) had no pituitary adenoma in pathology, and for 14 (21.5%) patients we did not have available pathology reports. Forty-six of the 65 (70.8%) patients experienced hormonal remission following surgery (Table 1).

During follow-up, 17 (26.2%) patients underwent repeated pituitary surgery, 8 of them due to persistent disease, and 9 due to recurrent elevated cortisol. Fifteen (22.1%) patients underwent radiation therapy. Thirty-nine (57.4%) patients received medical therapy for CD with adrenal steroidogenesis inhibitors and medications targeting pituitary somatostatin or dopamine receptors. Of these, seven (17.9%) patients were treated medically before TSS, 31 (79.5%) after TSS, and one (2.6%) patient received medical therapy both before and after TSS (Table 1).

### Abdominal imaging findings in patients with CD

Sixty-three patients had abdominal CT, including five who had Ga68 positron emission tomography (PET) CT without pathologic adrenal Ga68 uptake on functional imaging. Five additional patients had abdominal MRI.

Twenty-three (33.8%) patients underwent adrenal imaging as part of their medical evaluation following the diagnosis of Cushing’s syndrome. Five (7.4%) patients underwent adrenal imaging due to persistent or recurrent disease following surgery. Eleven (16.2%) patients had adrenal imaging because of abdominal pain, thirteen (19.1%) patients for other reasons not related to Cushing’s syndrome (e.g. following abnormal finding in ultrasonography (US), or before abdominal/gynecological surgery) and sixteen patients (23.5%) for unknown reasons.

**Fig. 2:**
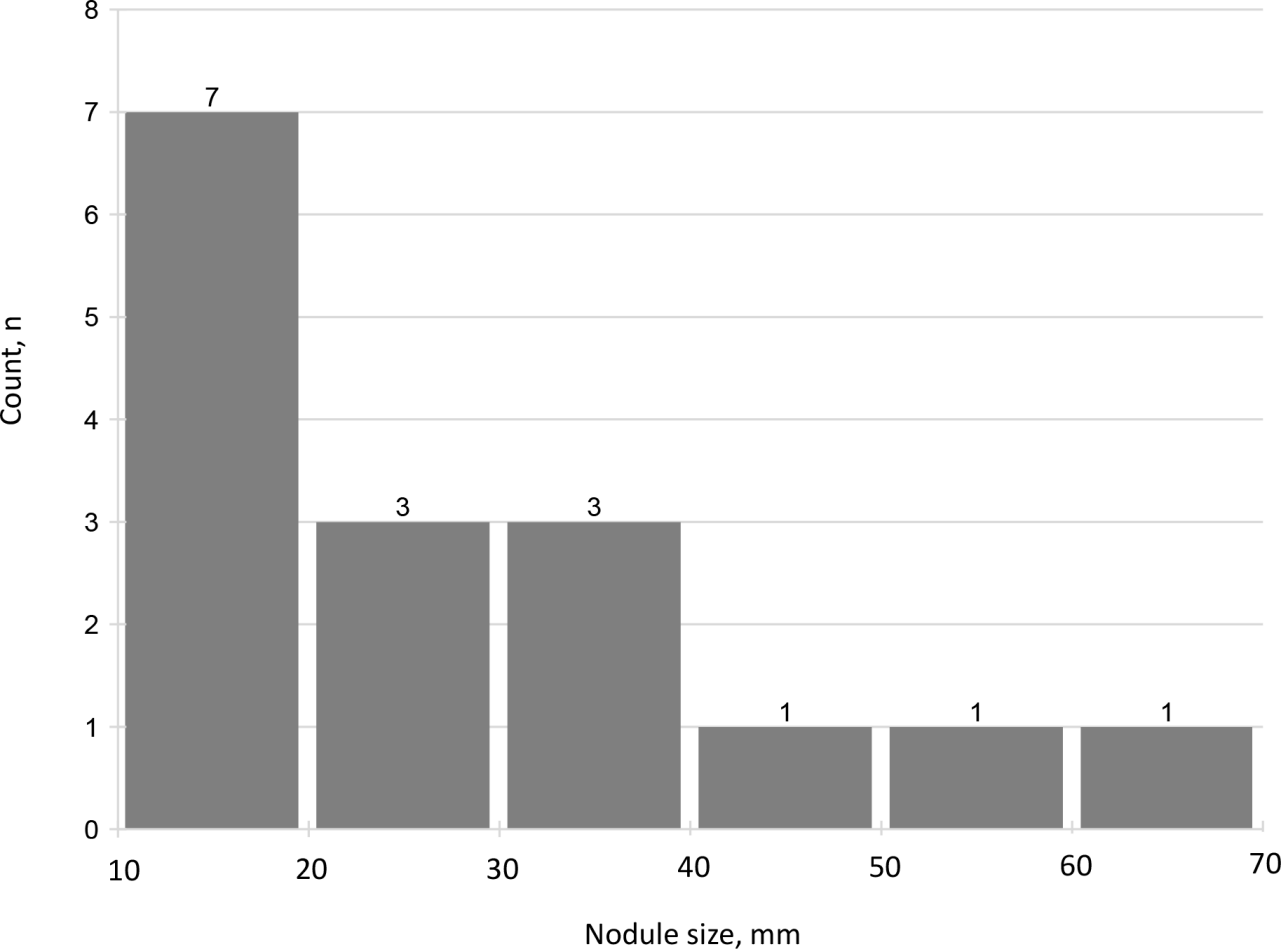
The size distribution of adrenal nodules (≥10 mm)

A total of 16 (23.5%) patients had an adrenal nodule ≥10 mm (median size, 27.5 mm, IQR 14.3–38.3) (Table 1). All of the nodules were radiologically defined as compatible with adenomas, based on low Hounsfield Units (HU) on non-contrast CT or signal drop on out-of-phase MRI sequences. Five patients had nodules in the right adrenal gland, six others had nodules in the left adrenal gland, and five patients had adrenal nodules ≥10 mm in both adrenal glands – one nodule in each side. There was only one patient with adrenal imaging consistent with bilateral multinodular adrenal hyperplasia, that was classified into the group of patients with adrenal nodule ≥10 mm. Nine (13.2%) patients had an adrenal nodule ≥20 mm (Fig. 2).

Nineteen (27.9%) patients had adrenal hyperplasia and/or nodules smaller than 10 mm (Table 1).

Patients with adrenal nodules ≥10 mm, as well as those with adrenal hyperplasia and/or nodules <10 mm, were significantly older compared to individuals with normal adrenal glands (mean age at imaging: 49.0 ± 12.4 and 50.4 ± 13.7 vs 39.1 ± 14.9 years, respectively; *p* = 0.03 and *p* = 0.01). Only patients with adrenal nodules ≥10 mm had significantly lower ACTH levels compared to patients with normal adrenal glands (0.7 x ULN, IQR 0.5–1.2, vs 1.2 x ULN, IQR 0.9–1.8; *p* = 0.02) (Table 2).

**Table 2:** Clinical characteristics of CD patients with adrenal nodules ≥10 mm, **adrenal hyperplasia and/or nodule <10** **mm** and with normal adrenal glands

Variable	Adrenal nodule ≥10 mm (*N* = 16)	Adrenal hyperplasia and/or nodule <10 mm (*N* = 19)	Normal adrenal glands (*N* = 33)	p-value (nodule ≥10 mm Vs normal adrenal glands)	p-value (hyperplasia and/or nodule <10 mm Vs normal adrenal glands)
Age at CT/MRI scan, years – mean (SD)	49.0 (12.4)	50.4 (13.7)	39.1 (14.9)	0.03	0.01
Pituitary adenoma size in mm, median (IQR)^a^	7.0 (6.0–11.0)	5.5 (3.0–13.0)	6.0 (4.0–8.0)	0.12	0.92
ACTH level x ULN, median (IQR)^b^	0.7 (0.5–1.2)	1.4 (0.9–2.2)	1.2 (0.9–1.8)	0.01	0.62
UFC level x ULN, median (IQR)^c^	4.0 (1.8–6.0)	3.8 (3.0–6.0)	3.5 (2.0–6.0)	0.77	0.63
Remission after TSS, n (%)^d^	12 (80%)	11 (61.1%)	23 (71.9%)	0.73	0.53

*ACTH* adrenocorticotropic hormone, *CD* Cushing’s disease, *CT* computed tomography, *MRI* magnetic resonance imaging, *UFC* urinary free cortisol, *ULN* upper limit of normal, *TSS* transsphenoidal surgery

^a^n 13/16, 14/19 and 27/33

^b^ n 12/16, 17/18 and 25/33

^c^n 13/16, 18/18 and 27/33

^d^n 12/15, 11/18 and 23/32

**Fig. 3:**
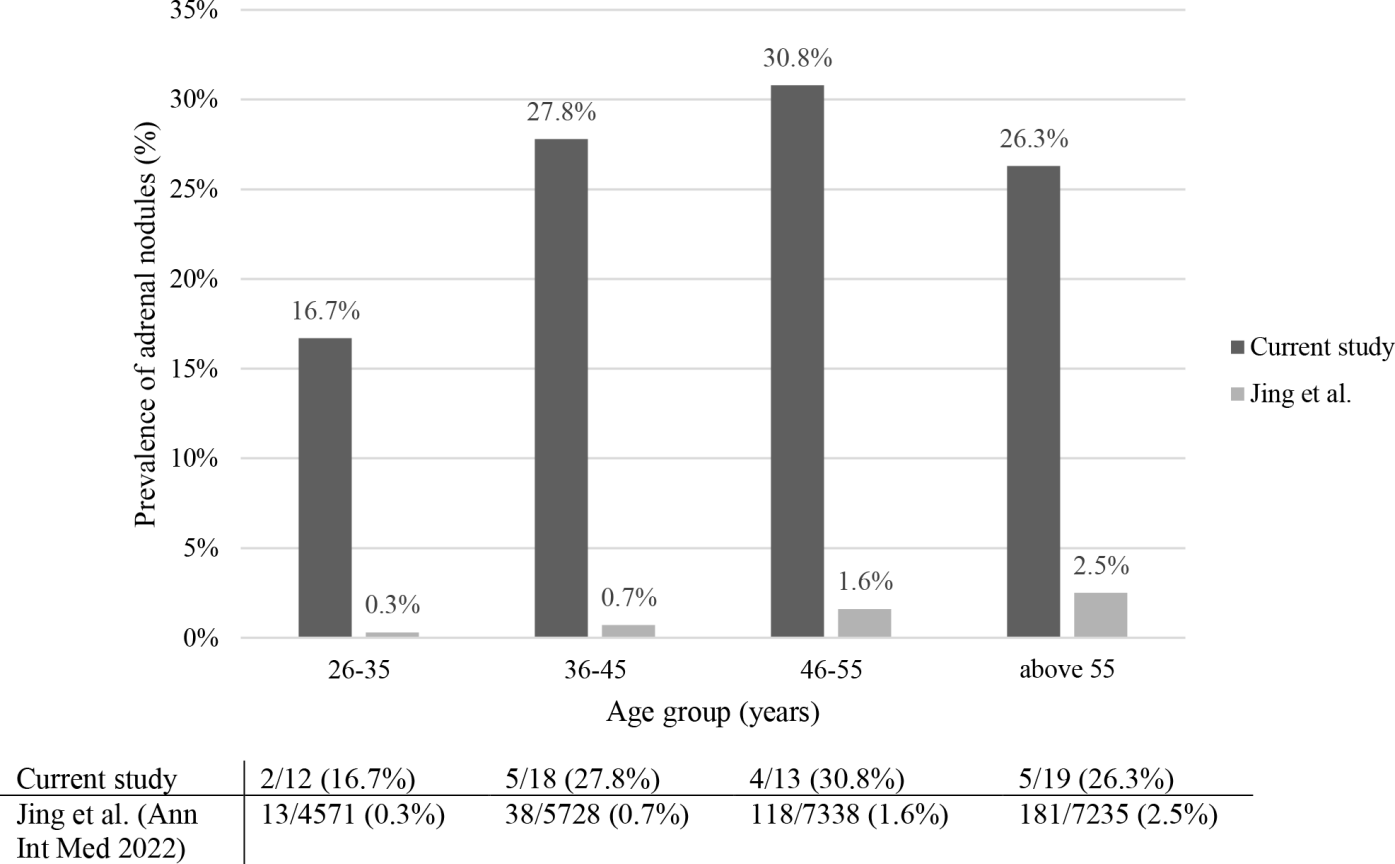
Prevalence of adrenal nodules ≥10 mm in CD patients (current study) and in the general population (Jing et al.), stratified by age group

The prevalence of adrenal nodules increased with age from 16.7% in patients aged 26–35 years to 27.8, 30.8 and 26.3% in those aged 36–45, 46–55, and above 55 years, respectively (Fig. 3).

Urinary free cortisol (UFC) levels did not differ significantly among the three groups. Patients with adrenal nodules ≥10 mm had a median UFC level of 4.0 x ULN (IQR 1.7–6.3), those with adrenal hyperplasia and/or nodules <10 mm had a median of 3.8 x ULN (IQR 3.0–6.0), and patients with normal adrenal glands had a median of 3.5 x ULN (IQR 2.0–6.0) (*p* = 0.77 and *p* = 0.63, respectively vs. normal adrenal glands) (Table 2).

A pituitary adenoma was depicted by sellar MRI in 54 (79.4%) patients. Patients with adrenal nodules ≥10 mm tended to harbor a larger pituitary adenoma compared to patients with normal adrenals (median adenoma size, 7.0 mm (IQR 6.0–11.5) vs 6.0 mm (IQR 4.0–8.0) respectively; *p* = 0.11). Patients with adrenal hyperplasia and/or nodules <10 mm had a median adenoma size of 5.5 mm (IQR 3.0–13.0), which was not significantly different compared to patients with normal adrenal glands (*p* = 0.63) (Table 2).

Remission rates after TSS were not significantly different among the groups. Among patients with adrenal nodules ≥10 mm, 12 (80%) patients achieved remission, compared to 23 (71.9%) patients with normal adrenal glands (*p* = 0.73). Similarly, 11 patients (61.1%) with adrenal hyperplasia and/or nodules <10 mm achieved remission, with no significant difference compared to patients with normal adrenal glands (*p* = 0.53) (Table 2).

### Clinical characteristics of four patients with CD who underwent unilateral adrenalectomy

Four patients (all females; mean age at the time of adrenal imaging, 46.3 ± 6.8 years) underwent unilateral adrenalectomy for a benign adrenocortical adenoma (adrenal adenoma size between 2.8–6.4 cm). One patient underwent adrenalectomy prior to TSS, with persistent hypercortisolism following adrenal surgery but achieved remission after TSS (Table 3).

**Table 3:** Clinical characteristics of four patients with CD who underwent unilateral adrenalectomy

Variable	Patient 1	Patient 2	Patient 3	Patient 4^a^
Gender (F/M)	F	F	F	F
Age at adrenal imaging (years)	40	55	42	48
Adrenal adenoma size (mm)	40	64	39	28
Pituitary adenoma diameter (mm)	6	12	6	5
Pre-adrenalectomy ACTH level x ULN	1.1	0.6	0.3	0.6
Post-adrenalectomy ACTH level x ULN	1.7	NA	NA	0.3
Pre-adrenalectomy UFC level x ULN	3	1.9	6.1	1.5
Post-adrenalectomy UFC level x ULN	NA	0.4	0.2	1.6
Remission after TSS (Yes/No/Transient)	Transient	Transient	No	Yes
Remission after unilateral adrenalectomy (Yes/No/Transient)	Transient	Yes	Yes	No

*F* female, *M* male, *ACTH* adrenocorticotropic hormone, *UFC* urinary free cortisol, *NA* not available, *ULN* upper limit of normal, *TSS* transsphenoidal surgery

^a^**Patient 4 –** initially underwent unilateral adrenalectomy without remission, followed by TSS which resulted in remission

Three patients underwent adrenalectomy after TSS. All three patients exhibited persistent hypercortisolism prior to adrenalectomy, with a median UFC level of 3.0 x ULN (IQR 1.9–6.1). The median ACTH level was 0.6 x ULN (IQR 0.3–1.1). One of them experienced transient postoperative cortisol normalization, while the other two achieved remission following adrenalectomy (Table 3).

### Abdominal imaging findings in patients with ACTH-dependent Cushing’s syndrome without tumor localization

Ten patients (including 7 females) with ACTH-dependent Cushing’s syndrome without tumor localization had adrenal imaging. Compared to patients with CD, these patients were older (mean age at the time of adrenal imaging, 58.4 ± 15.1 vs 44.7 ± 14.9 years, *p* < 0.01). Three of these patients (30.0%) had adrenal nodule ≥10 mm (median size, 14 mm, IQR 11.7–18), and 6 (60.0%) had adrenal hyperplasia or small nodules <10 mm (Table 1).

## Discussion

In the current study, we assessed the prevalence of adrenal nodular lesions and hyperplasia in patients with CD. We found that among 68 patients with CD who underwent adrenal imaging, 16 (23.5%) patients had an adrenal nodule ≥10 mm and 19 (27.9%) patients had adrenal hyperplasia or a nodule <10 mm. Additionally, we studied 10 patients with ACTH-dependent Cushing’s syndrome without tumor localization. Among these patients, 3 (30.0%) patients had an adrenal nodule ≥10 mm.

ACTH has trophic and mitogenic effects on the adrenal cortex [5], and chronic ACTH secretion may lead to adrenal hyperplasia. However, most of the literature on the co-existence of CD and adrenal nodules is based on case reports [16–18], with only a few studies focusing on the prevalence of adrenal nodules in cohorts of patients with CD [6, 7, 15].

Sohaib et al. reported that among 40 patients with CD, 25 (62.5%) had enlarged adrenal glands by CT, and seven patients (17.5% of the CD cohort) had an adrenal nodule ≥10 mm [7]. Imaki et al. found that among 24 patients with CD, 12 (50.0%) had adrenal hyperplasia, and only one (4.2%) had an adrenal nodule ≥10 mm [6]. Albiger et al. also studied the prevalence of adrenal nodules in CD, but defined a nodule as ≥5 mm. In their study, 15 out of 41 patients (36.6%) had adrenal nodules of this size [15] (Table 4). To the best of our knowledge, our study represents the largest investigation to date on the prevalence of adrenal abnormalities in CD patients (Table 4).

**Table 4:** Adrenal morphology in CD patients in the current study and three other main cohorts

Variable	Current study	Sohaib et al. (Am J Reont [7])	Imaki et al. (Endocrine J [6])	Albiger et al. (J Endo Inv [15])
CD patients - n	68	40	24	41
Female – n (%)	51 (75%)	30 (75%)	18 (75%)	NA
Age, years - mean (SD)	44.7 (14.9)	47 (11–73)^a^	43.0 (13.5)	39.9 (12)
Normal adrenal glands – n (%)	33 (48.5%)	15 (37.5%)	11 (45.8%)	16 (39.0%)
Adrenal nodule – n (%)	16 (23.5%)^b^	7 (17.5%)^b^	1 (4.2%)^b^	15 (36.6%)^c^
Adrenal hyperplasia and/or nodule <10 mm – n (%)	19 (27.9%)	18 (45%)	12 (50.0%)	10 (24.4%)

*CD* Cushing’s disease, *NA* not available

^a^Median age

^b^Adrenal nodule ≥10 mm

^c^Adrenal nodule≥5 mm

In our study all patients underwent adrenal imaging during the active phase of their disease. Notably, 28 (41.2%) patients underwent adrenal imaging as part of their medical evaluation following the diagnosis of Cushing’s syndrome or due to persistent or recurrent disease. Shoaib et al. included CD patients who underwent CT imaging as part of their radiological assessment [7]. Albiger et al. included CD patients who had undergone abdominal CT scan as part of their initial evaluation or the assessment for persistent or recurrent disease [15].

With the increased use of abdominal imaging in recent decades, incidental findings of abnormal adrenal lesions have become more common [8]. The prevalence of AIs in the general poppulation has been reported to range from 1.2–5.0% in various studies [8, 10–14, 19], which is significantly lower than the prevalence observed in our CD patients.

A large retrospective cohort from the United Kingdom, including 479,975 outpatients that underwent in-hospital CT or MRI scans (excluding patients with known adrenal lesion), found that 1.2% of individuals had AI [19]. The prevalence of AI was higher in patients who underwent abdominal CT imaging, reaching to 3.0%. The authors found a correlation between age and the prevalence of AI, ranging from 0.2% in the youngest group (21–30 years) to 4.1% in the oldest group (age ≥91 years) [19]. Consistent with findings from previous studies [7, 15], our study also demonstrates that patients with adrenal nodules were significantly older compared to those with normal adrenal glands.

A recent study from China, which examined 25,356 healthy individuals (unselected population) who underwent abdominal CT imaging, found that 351 (1.4%) had an adrenal tumor [13]. Compared to the results of this study, we observed that in each age group, CD patients had a much higher prevalence of adrenal nodules: between 26–35 years 16.7% in our cohort vs 0.3% in the Chinese population, between 36–45 years 27.8% vs 0.7%, for subjects 46–55 years 30.8% vs 1.6%, and above the age of 55 years 26.3% vs 2.5% (Fig. 3).

We found that patients with adrenal nodules≥10 mm had a significantly lower ACTH level. Albiger et al. also found that ACTH levels were significantly lower in patients with adrenal nodules. Their hypothesis was that a gradual transition from pituitary to adrenal autonomy might suppress ACTH production [15]. Previous reports have suggested that, in a subgroup of patients with prolonged ACTH stimulation, there might be a transition from pituitary dependent to adrenal dependent Cushing’s syndrome [20, 21]. Tabarin et al., found that dexamethasone suppressibility and the stimulatory effect of metyrapone on ACTH secretion were less in CD patients with hyperplasia and adrenal nodules than in those with diffuse adrenal hyperplasia, suggesting a greater degree of adrenal autonomy in the former [22]. Dalmazi et al., found somatic mutations in the gene encoding the catalytic *α* (C*α*) subunit of protein kinase A (PKA; PRKACA) in adrenal nodules of two patients with long-standing CD [23]. PRKACA somatic mutations are the most common genetic finding in adrenal adenomas associated with ACTH-independent Cushing syndrome [24], therefore these genetic alterations could represent a possible mechanism underlying adrenal nodule formation and autonomous cortisol hyperproduction in a subgroup of patients with long-standing CD.

In our study, 4 patients with adrenal nodules underwent unilateral adrenalectomy, two of them achieved full remission after the surgery. Interestingly, Dalmazi et al., described also that a patient who underwent unilateral adrenalectomy of a 35 mm adrenal nodule, achieved clinical and biochemical remission [23]. It may be worthwhile to consider unilateral adrenalectomy in selected CD patients with persistent hypercortisolemia after TSS, who have a large unilateral adrenal nodule.

There are several limitations to this study, primarily due to its retrospective design. The study cohort consists of patients who were referred for follow-up at endocrinology departments within tertiary hospitals. This setting likely leads to a higher frequency of imaging studies compared to the general population, potentially leading to a higher rate of incidental adrenal findings. Most of the patients did not have repeated adrenal imaging during their follow-up, so it is not possible to assess whether there was a change in the appearance of the adrenal glands following disease remission.

Moreover, there were instances where sufficient clinical data was unavailable to definitively confirm a diagnosis of CD in some patients. Thus, 10 patients were excluded from the main cohort analysis.

Another limitation is the relatively small sample size of the study population, which resulted in some findings not reaching statistical significance.

In conclusion, our study found that abnormal adrenal imaging was present in 51.5% of patients with CD. Notably, the prevalence of adrenal nodules in our cohort is 10 times higher than in the general population across all age groups, emphasizing a marked difference of adrenal morphology between CD patients and healthy individuals and suggesting that chronic ACTH stimulation leads to adrenal nodule development. The relative low levels of ACTH in patients with adrenal nodules may reflect partial autonomous cortisol secretion in some adrenal nodules. In light of this, adrenal nodules in patients with CD appear to be a relatively common finding, highlighting the importance of thorough laboratory and imaging diagnosis to identify the cause of hypercortisolism.

## Data Availability

The data that support the findings of this study are available from the corresponding author upon reasonable request.
